# Convergence Circuit Mapping: Genetic Approaches From Structure to Function

**DOI:** 10.3389/fnsys.2021.688673

**Published:** 2021-06-21

**Authors:** Jang Soo Yook, Jihyun Kim, Jinhyun Kim

**Affiliations:** ^1^Center for Functional Connectomics, Korea Institute of Science and Technology (KIST), Seoul, South Korea; ^2^Department of Integrated Biomedical and Life Sciences, Graduate School, Korea University, Seoul, South Korea

**Keywords:** viral tracers, optogenetics, chemogenetics, genetically encoded calcium indicators, genetically encoded transmitter indicators, synapse, brain mapping

## Abstract

Understanding the complex neural circuits that underpin brain function and behavior has been a long-standing goal of neuroscience. Yet this is no small feat considering the interconnectedness of neurons and other cell types, both within and across brain regions. In this review, we describe recent advances in mouse molecular genetic engineering that can be used to integrate information on brain activity and structure at regional, cellular, and subcellular levels. The convergence of structural inputs can be mapped throughout the brain in a cell type-specific manner by antero- and retrograde viral systems expressing various fluorescent proteins and genetic switches. Furthermore, neural activity can be manipulated using opto- and chemo-genetic tools to interrogate the functional significance of this input convergence. Monitoring neuronal activity is obtained with precise spatiotemporal resolution using genetically encoded sensors for calcium changes and specific neurotransmitters. Combining these genetically engineered mapping tools is a compelling approach for unraveling the structural and functional brain architecture of complex behaviors and malfunctioned states of neurological disorders.

## Introduction

Information is processed in the brain by a vast number of diverse neurons that are extensively intermingled and interconnected mainly through synapses. Structural and functional analyses of neural circuits are essential for deciphering the operational principles of the brain. Neurons often convey multiple layers of information converging from various cell types and/or brain areas, with integration of these signals coordinating a variety of behaviors and cognitions. To understand highly regulated and complex information processing in neural networks, knowledge of afferent input organization is crucial because functions of certain neurons within a brain region are shaped by the profiles of their inputs as initial signals. Therefore, increasingly, scientific endeavors are ongoing to untangle convoluted neural circuits at different levels (synapse–neuron–region) in various organisms (White et al., 1986; Stephan et al., 2001; Kötter, 2004; Sporns et al., 2005; Bock et al., 2011; Briggman et al., 2011; Van Essen et al., 2013; Oh et al., 2014; Hildebrand et al., 2017; Cook et al., 2019; Scheffer et al., 2020), generally starting with connections between defined populations of neurons and cell types (Druckmann et al., 2014; Kasthuri et al., 2015; Iascone et al., 2020). Developing and improving new technologies for identifying, labeling, imaging, and manipulating individual neurons, as well as populations of neurons in the context of behaviors, will lead to a comprehensive picture of neural network organization (Micheva and Smith, 2007; Kim et al., 2011; Yizhar et al., 2011; Ragan et al., 2012; Sternson and Roth, 2014; Lin and Schnitzer, 2016; Kornfeld and Denk, 2018). The initial step in these connectivity research studies relies on genetic engineering: well-established animal models expressing genetic switches, such as Cre and tetracycline-controlled transactivator, for defining and labeling certain populations and types of neurons (Sauer and Henderson, 1988; Gossen and Bujard, 1992; Gong et al., 2007; Gerfen et al., 2013; Harris et al., 2014, 2019; Hooks et al., 2018); viral systems with different features, such as tropism and axonal transduction, in a genetic switch-dependent manner (DeFalco et al., 2001; Wall et al., 2010; Lo and Anderson, 2011; Ährlund-Richter et al., 2019; Lazaridis et al., 2019; Sun et al., 2019; Gong et al., 2020); fluorescent sensors for monitoring neural events, such as intracellular Ca^2+^ influx and neurotransmitter release in response to neural activity (Lin and Schnitzer, 2016; Sabatini and Tian, 2020); and actuators for manipulating neuronal activity, such as opto- and chemo-genetics (Deisseroth, 2015; Roth, 2016; Atasoy and Sternson, 2018). The next step requires advances in imaging and reconstruction of genetically defined neural populations, and in sophisticated behavioral measurements linking the wiregram and dynamics of neural circuits to behaviors (Ragan et al., 2012; Gong et al., 2016; Harris et al., 2019; Wang et al., 2020).

The field of neuroscience has been rapidly and dramatically changing in the last two decades, fueled by innovations in molecular genetic engineering, imaging, and computer science. Recently equipped with advanced tools including microscopes and optrodes (Flusberg et al., 2008; Ghosh et al., 2011; Cui et al., 2013; Kim C.K. et al., 2016; Sofroniew et al., 2016; Zong et al., 2017; Skocek et al., 2018; Sych et al., 2019), researchers are increasingly capable of monitoring and perturbing the activity of specific types and populations of neurons as well as dissecting complex neural connectivity. It is no exaggeration to state that at the core of these advanced versatile tool boxes there are genetic tools making it possible to deliver tailored detectors, sensors, and manipulators to specific circuits, neurons, and synapses for studying the convergence of multiple information in the brain. Here, with particular attention to genetic approaches, we review the advanced tools for structural and functional mapping of convergence connectivity now available to neuroscientists working in the mouse. Beginning with a brief technical account and discussion of the unique significance of these approaches, we then discuss recent advances in their applications and combinatorial strategies for exploring structural and functional organization of various circuits.

### Structural Input Convergence Mapping With Anterograde Viral Tracers

**FIGURE 1:**
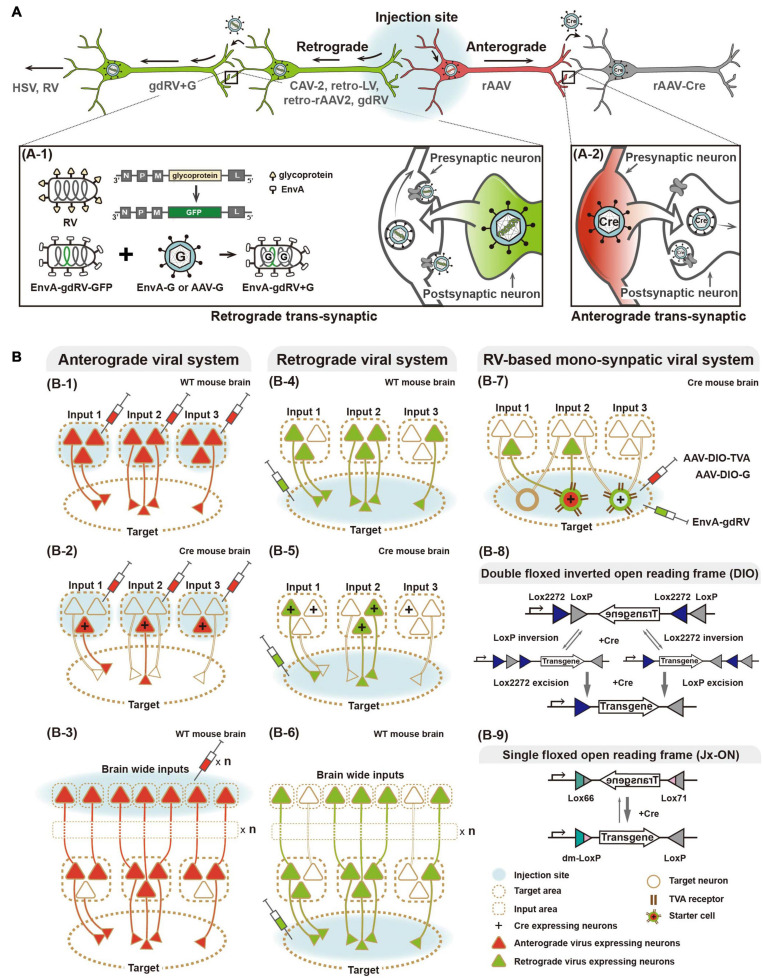
Viral systems for visualizing input convergent circuitry and delivering genetic tools for functional input mapping. **(A)** Schematic illustration of antero-, retrograde, and transsynaptic viral tracers. Anterograde virus (red) and retrograde virus (green) are distinguished by their neural entry site and axonal transport directions. Several viruses can cross synapse(s) and enter the connected neurons. Transsynaptic delivery of retrograde virus and engineered rabies virus, as an example of trans-synaptic retrograde viral tracers, are illustrated (A-1). Anterograde transsynaptic delivery of AAV 1 or 9 expressing Cre is illustrated (A-2). **(B)** Schematic illustration of labeling strategies of various viral systems. For convergence circuit mapping, anterograde virus to the multiple input areas (B-1–3) or retrograde virus to a target area (B-4–6) can be injected. Engineered rabies virus (RV) labels convergent circuits to the cell type neuron in the target area (B-7). Furthermore, antero- and retrograde polysynaptic viruses enable identifying neural circuitries integrated cross multiple synapses by injecting input and target areas, respectively (B-3,6). Two schematic strategies of Cre-dependent viral vectors for labeling particular cell types, such as Double floxed inverted open reading frame (DIO) and single floxed version (Jx-ON) (B-8,9).

A plethora of neuronal input studies have emerged from the idea that complex brain functions are operated by signal integration of various inputs and coordination of neural activity at the network level. One straightforward approach has been labeling of a certain neural population in a brain area with anterograde tracers and examination of the labeled axonal projections (Hunnicutt et al., 2014, 2016; Oh et al., 2014). Collective data from separate experiments labeling various neural populations, maximally two or three at once, illustrated convergence inputs. Owing to limitations of classical non-viral tracers, such as biotinylated dextran amine, we have focused on recombinant adeno-associated virus (rAAV) as the most commonly chosen anterograde viral tracer. Several reviews have extensively covered the limitations of conventional non-viral tracers (Nassi et al., 2015; Saleeba et al., 2019). In fact, synergetic application of anterograde AAVs expressing fluorescent proteins (FPs), advanced imaging techniques, and computational methods, have recently generated brain-wide and large-scale connectivity data that provide a comprehensive convergence map (Ragan et al., 2012; Hunnicutt et al., 2014, 2016; Oh et al., 2014; Kuan et al., 2015; Winnubst et al., 2019; Wang et al., 2020; Figure 1).

#### Anterograde Viral Systems

Viral vectors are a powerful tool for gene delivery in the nervous system, and in particular, rAAV is proven as an optimal gene delivery system with safety, efficiency, and practical ease (Wang et al., 2014; Haggerty et al., 2020; Xu et al., 2020). Given the technical advantages of rAAV, recent studies of input mapping have used rAAV-expressing FPs as reliable anterograde tracers. For instance, rAAV-expressing EGFP has been stereotaxically injected into various brain areas (Hunnicutt et al., 2014, 2016; Oh et al., 2014). Advances in imaging and computer capabilities made a standardized data generation and processing platform possible, thereby axonal projections were digitally traced throughout the whole mouse brain. These detailed high-resolution images and anatomically defined input/target areas are accessible in online resources (i.e., Allen Brain Map^1^). The Allen Mouse Common Coordinate Framework provides a fully digitalized three-dimensional (3D) average brain image. A labeled brain region space (Allen CCF v3 Brain Atlas) facilitates systematic analysis of input/output profiles throughout the whole brain. Taking advantage of these standardized and digitalized platforms, axonal projection datasets from various brain regions can be reconciled to generate a map of convergent circuitry, despite data being obtained through separate experimental performances. Furthermore, detailed descriptions of topographical patterns of these axonal projections to a target region (converged from various spatially and functionally segregated input areas) captures important information on the organization principles of neural underpinnings. These recent studies have delineated convergent circuitries suggesting rules of connectivity organization and functional domains, with particular attention to cortical inputs to the thalamus and striatum, thalamic nuclei inputs to the cortex, and thalamic inputs to the striatum.

Notably, there are interesting investigations using rAAV-mediated anterograde trans-synaptic tagging, although rAAV is generally known to be transduced via anterograde single-cycle infection rather than trans-neuronal/trans-synaptic transduction (Zingg et al., 2017, 2020). It has been demonstrated that rAAV can label trans-synaptic outputs via anterograde trans-synaptic transduction when packaged with serotype 1 and 9. AAV-mediated trans-synaptic labeling is restricted to Cre expression, and not other proteins of a similar size such as GFP, offering a faithful complementary set of tools for labeling trans-synaptic outputs. Its successful applications have been increasing in input-defined circuitry functions, such as cortico-collicular pathways for defense behaviors and motion discrimination (Zingg et al., 2017; Beltramo and Scanziani, 2019), and gustatory cortico–amygdala pathways for taste (Wang et al., 2018).

#### Cell Type-Specific Structure Mapping by Anterograde Viral Systems

Diverse cell types are believed to be responsible for specific functions in a given brain area. Thus, cell type-resolved connectivity profiling will undoubtedly provide novel insights into structural and functional organization of neural networks. Significant progress in classifying diverse neuronal cell types has been made through the development of new techniques, such as single cell RNA sequencing-based molecular profiling, and remains ongoing (Zeng and Sanes, 2017; Luo et al., 2018). Meanwhile, decades-long efforts together with advances in genomics and genetics have generated a useful resource of genetic switch driven lines (mainly Cre) in a cell type-specific manner (Gong et al., 2007; Gerfen et al., 2013; Harris et al., 2014). The rich collection of diverse Cre driver lines have made neuroscience research more efficient and precise, allowing cell type-specific labeling, monitoring, and manipulating. With respect to molecular identity, using these Cre driver lines, AAV engineered for Cre-dependent on/off switching modes has been widely used for selective labeling of a given neuronal type in a given brain area, such as projection pattern- and cortical layer-specific neurons. In the GENSAT collection^2^ for instance, two specific Cre drivers (Tlx3_PL56 and Sim1_KJ18) allow selective labeling of intra-telencephalic (IT)-type and pyramidal tract (PT)-type neurons by injection of AAV expressing Cre-dependent FPs in various sites of the sensory, motor, and frontal cortices (Hooks et al., 2018). This study revealed different topographic organization of cortico–striatal projections of IT- and PT-type neurons, suggesting that striatal regions integrate input convergence from multiple cortical areas via at least two different cell type-specific channels. Furthermore, using 49 different Cre driver lines to selectively label cell types in cortical layers showing different projection patterns, cortical cell type-specific connectivity mapping revealed hierarchically organized convergence in the thalamus (Harris et al., 2019).

### Structural Input Convergence Mapping With Retrograde Viral Tracers

Brain-wide mapping of multiple inputs converged into a given target area became evidently feasible and beneficial by collecting comprehensive information from individual datasets using anterograde virus data together with computational processing, as described above. However, anterograde virus-based convergence mapping can be incomplete, unless all multiple input areas and cell types are covered by viral labeling. Additionally, this approach requires careful validation because of potential misinformation delivered by ectopic injection of the anterograde tracer in neighboring and/or boundary regions around a given input area. Therefore, retrograde axonal transport of tracers, e.g., cholera toxin subunit B and rabies virus (RV), offers considerable advantages to selectively map multiple inputs to a subpopulation of neurons and even to a single neuron (Ugolini et al., 1987; Ugolini, 1995; Lanciego and Wouterlood, 2011; Zingg et al., 2014; Callaway and Luo, 2015; Junyent and Kremer, 2015; Tervo et al., 2016; Mandelbaum et al., 2019; Schwarz and Remy, 2019). In the past decade, there have been advances in developing and improving tools of retrograde viral systems for selective and precise input mapping. Again, several reviews have extensively covered the limitations of conventional non-viral retrograde tracers (Nassi et al., 2015; Saleeba et al., 2019).

#### Retrograde Viral Systems

Several types of virus, such as herpes simplex virus (HSV), canine adenovirus-2, and RV, are characterized by retrograde neurotropism through entry at axonal terminals followed by transport to cell bodies. With further engineering, retrograde viral systems have become a valuable tool for dissecting the structural and functional connectivity of various circuits (Norgren and Lehman, 1998; Soudais et al., 2003; Osakada et al., 2011; Kato et al., 2014; Kim E.J. et al., 2016; Tervo et al., 2016; Del Rio et al., 2019). Of these, the RV-based system offers specific retrograde access to mono-synaptically connected neurons to cell(s) in a brain region of interest (Wickersham et al., 2007b). RV is enveloped by a glycoprotein that mediates retrograde travel between synaptically connected neurons, possibly via multi-synaptic jumps. This feature makes it ambiguous to define directly connected pairs of neuronal populations. To construct a trans-synaptic tracer that travels retrogradely by only one synaptic step, a genetically engineered RV system has been developed (Mebatsion et al., 1996; Etessami et al., 2000; Barnard et al., 2006; Wickersham et al., 2007a,b). Glycoprotein-deleted RV (gdRV) is packaged with the envelope protein of avian sarcoma and leukosis virus (EnvA) to direct its entrance into so-called starter cells expressing avian tumor virus receptor A (TVA). EnvA-packaged gdRV is co-delivered to starter cells with a complementary virus expressing RV glycoprotein (e.g., AAV-G). In the starter cells, gdRV is further packaged with complementarity provided RV glycoprotein, and consequently spreads to presynaptic neurons by only one synaptic step. The engineered RV allows labeling of monosynaptic inputs to the starter cells in a cell type-specific manner—for instance, using Cre drivers and Cre-dependent strategies for expressing TVA (Wall et al., 2010). Given its advantages, such as unambiguous identification of synaptically connected neurons, RV-based systems have been increasingly used for structural and functional mapping of multiple inputs converging from various areas or cell types (Osakada et al., 2011; Dorocic et al., 2014; Sun et al., 2014, 2019; Tian et al., 2016; Ährlund-Richter et al., 2019; Lazaridis et al., 2019; Tasaka et al., 2020), as reviewed below. Owing to innate features of rabies virus system, cytotoxicity of rabies infection remains concerning in longer expression (Schnell et al., 2010; Callaway and Luo, 2015) and further effort to reduce its cytotoxicity is in progress (Reardon et al., 2016).

Alternatively, anterograde viral systems have been engineered for implementing a retrograde feature, while keeping their advantages such as a low immune response post-infection, low cytotoxicity, and stability. By engineering retrograde viral glycoproteins for pseudotyping (i.e., RV- and vesicular stomatitis virus-glycoprotein), variants of retrograde lentivirus (LV) have been developed and successfully applied in several animal models (Kato et al., 2011; Kato and Kobayashi, 2020). Using engineered retrograde LV, a recent study revealed action selection-related functions of the thalamo-striatal circuit in mice (Kato et al., 2018), and cognitive functions of prefrontal cortex networks in macaque monkeys (Oguchi et al., 2015).

Recently, as one of the most widely used and effective gene delivery systems, rAAV has been engineered for retrograde transport. Retrogradely accessible AAV, named rAAV2-retro, has been engineered by *in vivo* directed-evolution (Tervo et al., 2016), which is unlike intellectually designed strategies for retrograde LV pseudotyped with naturally existing retrogradely transportable glycoprotein. First, AAV vector libraries were constructed by packaging with engineered capsid variants generated by error-prone PCR, peptide insertion, randomization of loop regions, and DNA shuffling from wild-type capsid genes of variant serotypes. These virus libraries were then pooled and injected into the substantia nigra pars reticulata or cerebellum. Subsequently, spatially remote retrograde target tissues (such as the striatum or hindbrain, respectively) were collected and viral genomes extracted to select virions that had retrogradely reached these areas. Selected capsid sequences were re-cloned for the next AAV library that was subsequently evolved through iterative selection rounds. The final selected variant, rAAV2-retro, showed efficient retrograde access to projection neurons and sufficient expression of genetic tools, such as calcium sensors, for interrogations of structural and functional circuits. As cytotoxicity of RV remains an issue, rAAV2-retro provides promising potential and has been increasingly applied for various studies (Shang et al., 2019; Chen et al., 2020; Cushnie et al., 2020; Lafferty et al., 2020). More recently, another rAAV capsids have been engineered for retrograde transport (Davidsson et al., 2019; Düring et al., 2020). Davidsson et al. (2019) developed a method for capsid engineering, called barcode rational AAV vector evolution (BRAVE) combining rational design and direct evolution. New capsid variants (such as MNM004 and MNM008) generated by BRAVE have been demonstrated as a powerful tool for structural and functional connectivity studies. Düring et al. (2020) constructed self-complementary AAV-DJ/9 for retrograde transport in songbirds and mice by engineering the heparin binding domain that is important for cellular entry of virus (Grimm et al., 2008; Düring et al., 2020). Interestingly, these new engineered capsid variants show retrograde specific access to dopaminergic circuitry, which provides potential for not only various dopaminergic pathway-related studies but also clinical application.

#### Cell Type-Specific Structure Mapping by Retrograde Viral Systems

Similar to cell type-specific input mapping based on anterograde viral systems, retrograde viral tracers combined with genetic switch systems enable more detailed information of cell type-specific convergence connectivity. Sun et al. (2019) developed the Cre-dependent TVA-expressing mouse combined with the engineered monosynaptic-RV system to map synaptic inputs to specific cell types in the hippocampal CA1 region of TVA-expressing mice crossed with Cre drivers (CamK2a-, PV-, SOM-, and Dlx5/6-Cre for labeling excitatory pyramidal neurons, parvalbumin (+), somatostatin (+), and general interneurons, respectively). Additionally, recent studies described brain-wide mapping of mono-synaptically connected long-range inputs to different cell types of the medial prefrontal cortex using a Cre-dependent RV system with different interneuron-specific Cre drivers (PV-, SOM-, and VIP-Cre) (Ährlund-Richter et al., 2019; Sun et al., 2019). In Sun et al. (2019), RV-labeled input cells were further identified by immunostaining with several cell type markers, for example, cholinergic and serotonergic neurons. Similarly, another recent study described whole-brain mapping of glutamatergic inputs to the lateral habenula (LHb), and further identified RV-labeled input cell types by single nucleus RNA sequencing (snRNA-seq) (Lazaridis et al., 2019). To define glutamatergic inputs into the LHb, Cre-dependent gdRV-expressing EGFP was injected into the LHb of a vesicular glutamate transporter type 2 (vGluT2) Cre-line. LHb-projecting glutamatergic neurons in the globus pallidus internal segment and lateral hypothalamic area (LHA) were profiled by molecular analysis in terms of gene expression for GABA/glutamate co-releasing components as well as other cell markers. This study revealed that the glutamatergic LHA–LHb circuit is a critical node in value processing, with further functional assessments using activity actuators and sensors, which we review below. Furthermore, combinatorial anterograde and retrograde viral systems enable integration of cell type-specific inputs and target mapping (Dorocic et al., 2014).

### Functional Input Convergence Mapping With Activity Actuators and Sensors

**FIGURE 2:**
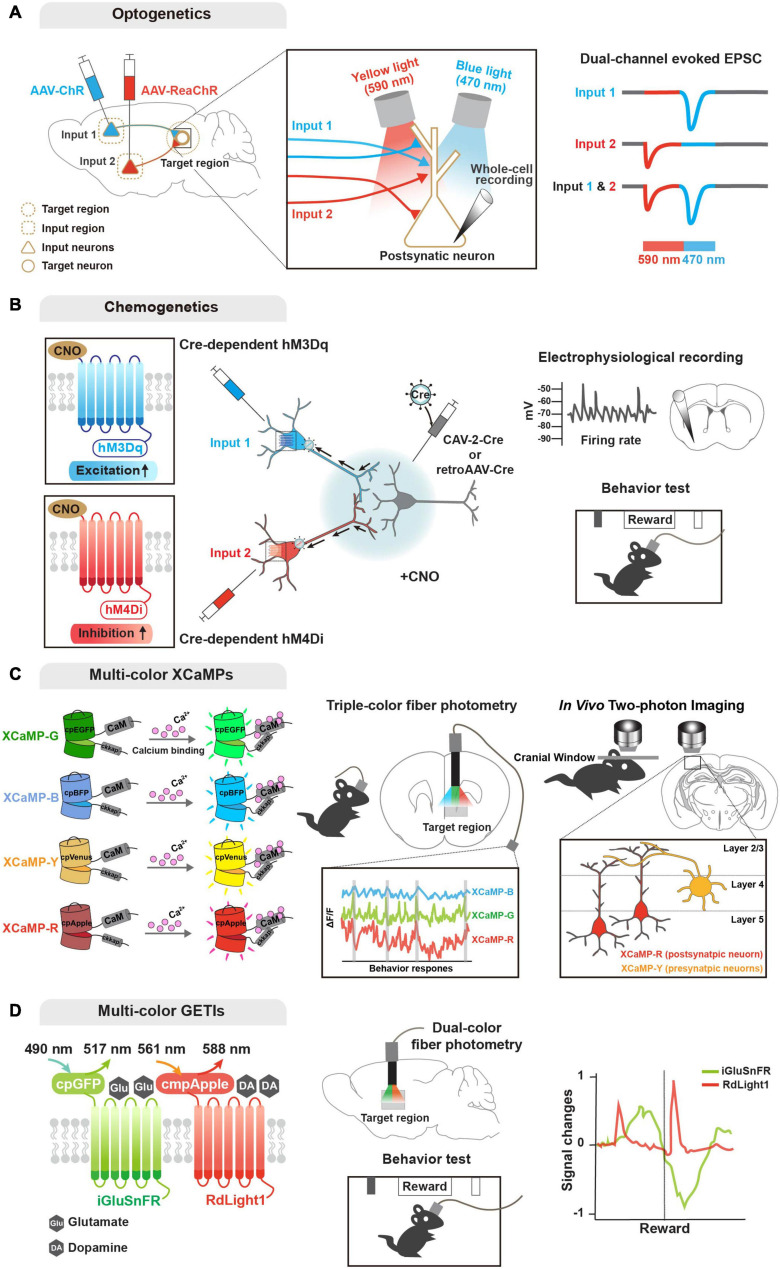
Genetic tools for functional input convergence mapping. **(A)** Schematic illustration of dual-channel ChR-assisted circuit mapping (2CRAM). AAV expressing ChR2 and ReaChR are injected in different input regions/cell types and postsynaptic target cell recording after illumination (sequential stimulation of 590 and 470 nm lights) represents functional convergence. **(B)** Combinatorial strategy of DREADD and retrograde virus for input-selective manipulation. Input neurons are labeled with retrogradely delivered Cre using CAV-2 or retroAAV virus and Cre-dependent DREADDs (e.g., hM3Dq and hM4Di). Electrophysiological recording or behavioral analysis (e.g., reward seeking) after CNO ligand administration represents functional convergence. **(C)** Illustration of multi-color GECIs for convergent circuit mapping. Multi-color GECIs (XCaMP-G, -B, -Y, and -R) consist of a circularly permuted FPs and enable to monitor different cell-type activities in the target region of the freely behaving mice. Dual-color imaging for XCaMP-labeled pre- and postsynaptic neurons at a target region represents convergent circuit. **(D)** Illustration of multi-color GETIs for convergent circuit mapping. Dual-color imaging for co-expressing iGluSnFR and RdLight1 at the target region enable to monitor glutamate and dopamine releases during behavior test (e.g., reward seeking).

So far, we have described antero- and retrograde viral systems expressing mainly FPs to visualize convergence of structural inputs. Combined with another set of genetically encoded tools for monitoring and manipulating neural activity, these useful viral systems have been applied to interrogate the functional significance of input convergence. Beginning with a brief technical account of recently developed manipulators and sensors of neural activity, we will review recent studies describing their combinatorial applications for physiological and behavioral studies as network-level phenomena (Figure 2).

#### Activity Actuators: Opto- and Chemo-Genetics

To dissect circuit functions underlying complex behaviors and various neurological diseases, an obvious approach is perturbation of neural activity in a specified circuit; this was traditionally performed by lesion studies and electrical stimulation. Through perturbation-triggered physiological, developmental, and behavioral alterations, potential causality between neural activity of specific circuits with brain functions and behaviors have been discovered. In past years, great advances have been made in manipulating neural activity at specific and precise spatiotemporal resolution using e.g., light- and chemical-controllable genetic tools (i.e., opto- and chemo-genetics, respectively). This has led to advances in circuit-level understanding of brain functions and diseases (Kim et al., 2017; Atasoy and Sternson, 2018).

Optogenetic approaches are based on light-sensitive microbial opsins, and enable activation and inactivation of neuronal activity by illumination that opens light-gated ion channels (Boyden et al., 2005; Zhang et al., 2007; Chow et al., 2010; Deisseroth, 2015). Early optogenetic molecules included channelrhodopsin-2 (ChR2) for activation and halorhodopsin for inhibition (Boyden et al., 2005; Zhang et al., 2007). ChR2 is a blue light-gated cation channel conducting H^+^, Na^+^, K^+^, and Ca^2+^ ions, and enabling action potential elicitation. Alternatively, halorhodopsin is a yellow light-gated chloride pump that enables membrane hyperpolarization. These optogentic tools can be delivered into a certain circuit or cell type (mostly by viral gene delivery), and then activated by a small and lightweight implanted fiber-optic probe (Adamantidis et al., 2007; Aravanis et al., 2007; Atasoy et al., 2008; Kuhlman and Huang, 2008), which is connected to a laser diode or light-emitting diode (LED) light source of different wavelengths. Further engineering generated variants of optogenetic tools in terms of light sensitivity, kinetics, functional stability, and so forth. Of the new opsins, red-shifted optogenetic variants (e.g., Crimson and Red-activable [ReaChR]) enable activation of neuronal populations by red light illumination (Zhang et al., 2008; Lin et al., 2013; Klapoetke et al., 2014), and offer the potential to explore circuitry functions of distinct neuronal populations by combinatorial yet specific excitations. In fact, a combinatorial strategy using ChR2 and ReaChR (named 2-channel ChR-assisted circuit mapping [2CRACM]) has been used for mapping functional convergence of multiple inputs from the primary somatosensory cortex and posterior medial thalamus on the primary motor cortex (Hooks et al., 2015; Prasad et al., 2020).

As a powerful chemo-genetic approach, designer receptor exclusively activated by designer drug (DREADD), is based on engineered ligand-sensitive receptors and an exogenous ligand specific for these receptors to in/activate neural activity (Sternson and Roth, 2014; Roth, 2016). DREADD utilizes engineered G protein-coupled receptors (GPCR) that respond exclusively to synthetic exogenous chemicals as a ligand, but not to their natural endogenous ligands. With a choice of Gq- and Gi-coupled GPCRs, DREADD can trigger activation and inactivation, respectively, of neuronal activity via intracellular signaling cascades. Similar to optogenetic tools, variants of DREADD have been developed e.g., mutated muscarinic acetylcholine (hM3Dq and hM4Di for activation and inactivation, respectively, by clozapine-N-oxide) and κ-opioid receptors (KORDi for inactivation of neuronal activity by salvinorin B) (Armbruster et al., 2007; Alexander et al., 2009; Vardy et al., 2015). In particular, a recent study demonstrated that two DREADD variants activated by different ligands, such as KORDi and hM3Dq, enable modulation of neuronal activity and further behaviors (Benekareddy et al., 2018). In parallel, improvement and development of new ligands with greater specificity and less potential side effects are underway. Compared with optogenetics, DREADD-based manipulation has pros and cons: (1) time; optogenetics offers high and precise temporal resolution (on the millisecond-scale), while DREADD-based manipulation is not elicited immediately and is prolonged (on the hour-scale). Nevertheless, this prolonged activation period by DREADD can be beneficial for behavioral and disease models. Furthermore, (2) invasiveness; DREADD offers a less invasive option and is more flexible with simple injection of ligands, while the optogenetic approach requires an implanted intracranial light source. Yet these neuronal activity actuators have become one of the most powerful tools for functional studies of various circuits (Smith et al., 2016; Campbell and Marchant, 2018; Luo et al., 2018; Lee et al., 2020). Moreover, various combinatorial strategies of optogenetics, DREADD, engineered viral systems, and cell type-specific Cre drivers have been designed and successfully applied for investigating functional convergence (Kato et al., 2018; Johansson and Silberberg, 2020; Lafferty et al., 2020; Prasad et al., 2020; Soden et al., 2020; Yamawaki et al., 2020; Zolnik et al., 2020).

#### Activity Sensors: GECI and GETI

Monitoring neuronal activity and synaptic events with precise spatiotemporal resolution is necessary to decipher functional information processing engaged in complex behaviors and malfunctioned states of neurological disorders. Development and refinement of genetically encoded sensors will permit better understanding of how neuronal dynamics is encoded in which neural circuits to control brain functions.

Regarding activity sensors (Lin and Schnitzer, 2016), genetically encoded calcium indicators (GECIs) provide the most mature modality for monitoring neural activity. As neural activity causes rapid changes in intracellular Ca^2+^ level, GECIs have advanced our knowledge of functional circuits (Broussard et al., 2014). GCaMP, the most enthusiastically used GECI, is an intracellular Ca^2+^ indicator comprising a circularly permuted FP (cpFP, typically cpGFP), Ca^2+^-binding protein calmodulin (CaM), and a Ca^2+^/CaM-sensing domain (typically, M13). Earlier GECI versions are based on Forster resonance energy transfer (FRET) of paired FPs. Single FP-based engineering has virtually revolutionized development of biosensors with sufficient brightness for *in vivo* events. In the presence of calcium, bright fluorescence can be detected through conformational changes triggered by Ca^2+^ binding to CaM, with poor fluorescence in the absence of calcium. GCaMP has evolved by engineering its variants to enhance the signal-to-noise ratio, sensitivity, and kinetics (Nakai et al., 2001; Tallini et al., 2006; Tian et al., 2009; Akerboom et al., 2009, 2012; Muto et al., 2011; Chen et al., 2013; Sun et al., 2013; Dana et al., 2016). Additionally, similar to the activity actuators described above, understanding of complex activity dynamics of convergent circuits demands multicolor availability of GECIs. Accordingly, recent efforts have succeeded in providing various colored GECIs that are applicable *in vivo*, including R-CaMP2, jRGECO1a, and XCaMP-Blue, -Yellow, and -Red (Zhao et al., 2011; Inoue et al., 2015, 2019; Dana et al., 2016).

Multicolor availability of GECIs has enabled simultaneous monitoring of neuronal activity of postsynaptic compartments innervated by multiple cell type-specific presynaptic neurons (Inoue et al., 2019). More recently, another expanded endeavor has developed genetically encoded indicators for imaging the release of specific neurotransmitters and neuromodulators. Inspired by the successful application of cpFP in the GCaMP family, various genetically encoded transmitter indicators (GETIs) have been developed using engineered ligand-binding proteins fused with cpFP that is conformationally changed upon binding of glutamate, GABA, acetylcholine, serotonin, and dopamine: iGluSnFR, iGABASnFR, GACh, iSeroSnFR, and dLight, respectively (Marvin et al., 2013, 2019; Jing et al., 2018; Patriarchi et al., 2018; Unger et al., 2020). Again, further development of color variants as well as optimization are ongoing (Wu et al., 2018; Patriarchi et al., 2020). GETIs can importantly dissect neural activity by detecting the release of various neurotransmitters and neuromodulators. Recent studies have demonstrated that application of multicolor GETIs combined with ChR2 and GECI can increase understanding of the functions and mechanisms of complex neural circuitry by mapping input convergence (Patriarchi et al., 2018, 2020; Kazemipour et al., 2019; Lee et al., 2021).

### Functional Input Convergence Mapping With Combinatorial Labeling Tools

A large variety of the powerful tools described above have enriched the neuroscientific arsenal by their combinatorial applications. Today strategies for labeling and defining a specific neuronal population underlying specific behavioral functions has become possible, yet increasingly complex, sophisticated, and representational. Combining active cell labeling, retrograde RV, and DREADD, a recent study revealed that the temporal association cortex (TeA) receives monosynaptic multiple inputs converging from cortical and subcortical areas, playing a critical role in auditory-driven maternal preference for pup calls (Tasaka et al., 2020). In this study, neuronal populations active in response to ultrasonic vocalizations (USVs) were determined by targeted recombination in active populations (TRAP), which is designed to express inducible Cre (CreERT2) under a control of an immediate early gene, such as Fos (Guenthner et al., 2013). Further combination of TRAP and RV allowed visualization of functional input connectivity by introducing RV specifically into TRAPed USV-responsive neurons of the TeA as starter cells. DREADD expression in these USV-TRAP neurons of the TeA demonstrated their functional link to a maternal behavioral effect.

In another recent study, Gong et al. (2020) demonstrated a clever and complex combinatorial strategy for convergence of feeding and drinking circuits, taking advantage of various advanced technologies, i.e., anterograde polysynaptic HSV, Fos-mapping, image segmentation using the standardized Allen Brain Atlas, optogenetics, and GECI. The recombinant H129 strain of HSV, a cell type-targetable version, has been demonstrated as an effective anterograde trans-synaptic viral tracer for labeling poly-synaptically connected output cells in cell type-specific Cre lines (Lo and Anderson, 2011). To broadly and unbiasedly search for a convergence hub-type region of feeding and drinking circuits, anterograde polysynaptic HSV was introduced into defined hunger-and-thirst-related neurons, such as Agouti-related protein (AGRP) neurons in the hypothalamic arcuate nucleus (ARC) and nitric oxide synthase 1 (Nos1) in the suprafornical organ (SFO). In addition to identifying active downstream areas engaged in hunger and thirst, Fos-immunostaining was performed after ontogenetic stimulation of these defined hunger-and-thirst-related neurons. The two datasets of cell type-specific HSV and Fos labeling were processed using the Allen Reference Brain for identifying hotspot areas as a convergence point. This intention map of HSV and Fos labeling guided further functional investigations which identified glutamatergic neurons in the peri-locus coeruleus as a polysynaptic convergence hub for hunger and thirst circuits.

### Convergence Mapping at the Synapse Level

Thus far, we have described various advanced tools for mapping structural and functional input convergence, mainly at the regional and cellular levels. Because the synapse is the primary unit of information processing, detailed synapse-level descriptions of connectivity converging from multiple individual neurons has strengthened our understanding of fine-scale organization of synaptic input profiles governing global and subcellular signal computations.

Electron microscopy (EM)-based dense mapping, with significant recent advances in large-scale image data acquisition and 3D volume reconstruction, allows visualization of relatively complete neuronal structures, offering high resolution on the nanometer ultrastructure scale (Briggman et al., 2011; Helmstaedter et al., 2013; Morgan et al., 2016; Schmidt et al., 2017; Motta et al., 2019). In recent studies, a combination of two-photon calcium imaging, optogenetics, and EM-based reconstruction permitted synapse-level functional convergence mapping (Bock et al., 2011; Briggman et al., 2011; Lee et al., 2016; Liang et al., 2018; Borges-Merjane et al., 2020). One such study used GECI, such as GCaMP6, to monitor the activity of retina ganglia cell (RGC) axons and dorsolateral geniculate nucleus (dLGN) neurons upon visual stimulation. This was followed by 3D EM reconstruction of dLGN dendrites innervated by RCG axonal boutons (Liang et al., 2018). Liang et al. (2018) demonstrated that clusters of boutons from different RGC axons on dLGN dendrites share similar visual feature preferences, and that one RGC axon can innervate multiple bouton clusters specialized for different visual feature preferences. As it is believed that dendritic signal processing is facilitated by spatially and temporally organized synaptic input patterns such as clustering (Baden et al., 2016; Gökçe et al., 2016; Wilson et al., 2016; Rompani et al., 2017), these results provide important details about the functional implication of fine-scale convergence for the transmission and integration of visual information from the retina to thalamus.

Additionally, recent studies have described fine-scale distributions of excitatory and inhibitory synaptic input convergence onto individual pyramidal neurons in different cortical layers by EM-based (Karimi et al., 2020) and fluorescent labeling of synaptic component-based 3D reconstruction (Iascone et al., 2020). Iascone et al. (2020) used genetically labeled pre- and post-synaptic components, such as Gephyrin-EGFP and Homer1c-tdTomato, respectively, as well as annotated spines to map E and I synapses. This study revealed local E/I balance in specific dendritic domains in layer 2/3 cortical neurons that might restrict dendritic and somatic firing. These detailed synaptic mapping studies reveal a precise excitatory/inhibitory balance suggesting distinct principles of signal integration in individual neurons. Using such fluorescent labeling of synaptic components, array tomography (AT) has been developed to visualize synaptic architecture and connection (Micheva and Smith, 2007; Micheva et al., 2010; Collman et al., 2015). AT is a combinatorial method for synaptic composition and connectivity mapping by reconstruction of images of serial ultrathin sections that can be labeled by immunofluorescence and imaged by fluorescence and EM. AT offers detailed synaptic compositions by repeated immunofluorescence labeling of multiple synaptic components.

Alternatively, for synapse-level connectivity mapping, genetically encoded synaptic detectors have been developed, and in particular, GFP reconstitution across synaptic partners (GRASP) technology. These are based on functional complementation between two non-fluorescent split-GFP fragments targeted to the synaptic membranes of the synaptic cleft (Feinberg et al., 2008; Gordon and Scott, 2009). Further variants of GRASP, such as mGRASP, eGRASP, tGRASP, and syb:GRASP, are available for improved accuracy, efficacy, and specificity to detect synapses in complex circuits (Kim et al., 2011; Macpherson et al., 2015; Choi et al., 2018; Shearin et al., 2018). Combination of mGRASP and optogenetics demonstrated that functional measures of synaptic strength correspond strongly with mGRASP-based structural measures of synapse size, which enabled high-resolution functional connection mapping (Song et al., 2018). Similar to the needs for red-shifted ChR and XCaMP, differently colored varieties known as X-RASPs (i.e., yellow Y-RASP and cerulean C-RASP) have been developed. This has broadened their utility to simultaneous labeling of synapses innervated by different inputs conveying distinct information, for instance, thermo-sensory and visual information (Macpherson et al., 2015) and engram and non-engram (Choi et al., 2018). These GRASP-based approaches can be expanded into multiple synapse-level convergence mapping when limits of color detection are overcome with further optimization and combination in sophisticated strategies.

## Conclusion and Future Perspectives

In this review, we have described advanced techniques, mainly genetic tools that are currently available for mapping anatomical and functional convergence connectivity. These tools mostly rely on imaging systems and computational platforms that are also in rapid progress (Lichtman et al., 2014; Zong et al., 2017; Kornfeld and Denk, 2018; Skocek et al., 2018; Sych et al., 2019; Wang et al., 2020). Creative and sophisticated combinations of all the above techniques are underway and will go a long way toward allowing untangling complex inputs in neural circuits. Further innovative new technologies are still required, such as less-toxic and definable trans-synaptic viral systems and genetic switches with reversible and spatiotemporally precise on/off control. Continuous upgraded versions of currently available tools and innovations of new tools joined with integrative and combinatorial approaches will provide deeper understanding of how multiple integrative information coordinates a variety of behaviors and cognitions.

## Author Contributions

JSY, JK, and JK wrote this manuscript. All authors contributed to the article and approved the submitted version.

## Conflict of Interest

The authors declare that the research was conducted in the absence of any commercial or financial relationships that could be construed as a potential conflict of interest.

## Footnotes

1 http://www.brain-map.org/

2 http://www.gensat.org
